# A new global gridded anthropogenic heat flux dataset with high spatial resolution and long-term time series

**DOI:** 10.1038/s41597-019-0143-1

**Published:** 2019-07-31

**Authors:** Kai Jin, Fei Wang, Deliang Chen, Huanhuan Liu, Wenbin Ding, Shangyu Shi

**Affiliations:** 1 State Key Laboratory of Soil Erosion and Dryland Farming on the Loess Plateau, Institute of Soil and Water Conservation, Northwest A&F University, Yangling, 712100 Shaanxi China; 2 Institute of Soil and Water Conservation, Chinese Academy of Sciences and Ministry of Water Resources, Yangling, 712100 Shaanxi China; 3 University of Chinese Academy of Sciences, Beijing, 100049 China; 4 Regional Climate Group, Department of Earth Sciences, University of Gothenburg, Box 460, 405 30 Gothenburg, Sweden; 5 College of Natural Resources and Environment, Northwest A&F University, Yangling, 712100 Shaanxi China

**Keywords:** Climate change, Energy and society

## Abstract

Exploring global anthropogenic heat and its effects on climate change is necessary and meaningful to gain a better understanding of human–environment interactions caused by growing energy consumption. However, the variation in regional energy consumption and limited data availability make estimating long-term global anthropogenic heat flux (AHF) challenging. Thus, using high-resolution population density data (30 arc-second) and a top-down inventory-based approach, this study developed a new global gridded AHF dataset covering 1970–2050 based historically on energy consumption data from the British Petroleum (BP); future projections were built on estimated future energy demands. The globally averaged terrestrial AHFs were estimated at 0.05, 0.13, and 0.16 W/m^2^ in 1970, 2015, and 2050, respectively, but varied greatly among countries and regions. Multiple validation results indicate that the past and future global gridded AHF (PF-AHF) dataset has reasonable accuracy in reflecting AHF at various scales. The PF-AHF dataset has longer time series and finer spatial resolution than previous data and provides powerful support for studying long-term climate change at various scales.

## Abstract (summary)

**Table:** 

Design Type(s)	modeling and simulation objective • time series design • data integration objective
Measurement Type(s)	climate change
Technology Type(s)	computational modeling technique
Factor Type(s)	anthropogenic generation of energy • Population Density
Sample Characteristic(s)	Earth (Planet) • anthropogenic habitat

Machine-accessible metadata file describing the reported data (ISA-Tab format)

## Background & Summary

Human activities have caused substantial changes to the global climate. In addition to the effects of greenhouse gases, aerosols, and land use/cover change, anthropogenic heat released from energy consumption can affect climatic changes at various scales^1–4^. For example, Zhang *et al*.^5^ found that energy consumption could lead to increases in winter and autumn temperatures of up to 1 °C in the mid- and high latitudes across North America and Eurasia. Ichinose *et al*.^1^ found that the maximum anthropogenic heat flux (AHF) in central Tokyo, Japan, was as high as 1,590 W/m^2^ in winter, resulting in warming to a maximum of 2.5 °C. Moreover, anthropogenic heat can affect wind speed because it reduces the stability of the boundary layer and enhances vertical mixing^6^. In view of the effects of anthropogenic heat on climate at local and continental scales and the increasing consumption of energy worldwide, the potential significance of anthropogenic heat as it relates to global climate change over a long-term period should be further studied using techniques such as global climate models.

The diversity of regional energy consumption and limited data availability make it difficult to produce high quality global AHF datasets when considering the accuracy^3^, resolution^7^, and length of time series^8^ of the data. For example, Allen *et al*.^9^ developed a widely accepted AHF dataset at 2.5 × 2.5 arc-minute resolution based on the Large Scale Urban Consumption of Energy model; however, it still cannot satisfy the demand of high-resolution regional modelling because of the relatively rough spatial resolution. Additionally, acquiring all of the necessary data (e.g., population, temperature, and different types of the energy sources) has proved to be impossible for many countries such as island countries^10^. Moreover, Chen *et al*.^3^ and Yang *et al*.^11^ estimated global AHFs based on the Defence Meteorological Satellite Program/Operational Linescan System (DMSP/OLS) night-time light data, but the night-time light data were normally limited by the short time series (1992–2013) available, “light diffusion” phenomena in densely populated areas^12^, and the differences in culture and day-length among different regions^13^. All of these limitations remained in producing AHF data and hindered simulation studies of anthropogenic heat at local, regional, or global scales. Remarkably, the time series of previous AHF datasets generally is not long enough to be used for simulation studies of long-term climate change (e.g., the dramatic global warming period since the 1980s).

Levels of energy consumption are expected to increase in the future because of the rapid development of the global economy and increased living standard. Therefore, it makes sense to study the variations and climatic effects of global AHF in the future. So far, only Flanner has projected the distribution of global AHFs in the future^2^. Flanner’s AHF dataset which was developed at 2009 included the global gridded AHF during 2005–2040 and in 2100; nevertheless, all of the data, except data for 2005, were projected data^2^. That is, Flanner’s AHF data during 2006–2040 were calculated based on the 2008 energy-use projection (high economic growth case) from the U.S. Energy Information Administration (EIA); his data for 2100 were calculated based on the assumption that energy consumption will grow by 2% per year for each country after 2040^2^. Evidently, Flanner’s data from 2006 to 2040 and the EIA energy-use projection in 2008 have been out of date; his assumption on energy consumption after 2040 should be improved due to the varied energy demands of each country^2^. Recently, a report of the McKinsey Global Energy Perspective (GEP) was proposed (https://gep.mckinseyenergyinsights.com/), which takes into account both macro- and micro-economic developments. A unique aspect of the GEP is that most insights on future energy demand are local (i.e., projecting the evolution of energy systems by country). This provides an opportunity to re-estimate the distribution of future global AHF.

The inventory-based approach is widely used to estimate anthropogenic heat release, including for both bottom-up and top-down approaches^14^. The bottom-up approach relies on detailed datasets of local land use and statistics on the hourly variation in energy consumption; this type of data is mostly used for local-scale studies such as a single city because of limited data availability^4,15^. In contrast, a top-down approach is often used for large-scale studies through allocating total energy consumption (e.g., energy consumption at country-level) into specific regions^7^. However, some detailed information about local energy consumption (e.g., industrial scale and distribution) is difficult to capture. Therefore, when using the top-down approach, some spatial data closely associated with energy consumption intensity such as population density and/or night-time light data are normally used as a proxy for the redistribute the total energy consumption^8^. The basic assumption of the top-down approach is that all energy consumed by human activities in a specified region is directly converted to anthropogenic heat in that region^14^. This assumption is somewhat insufficient because the transformation and storage of energy are ignored. However, detailed processes involved in heat emissions are not easily tracked in large-scale studies because transformation and storage can vary widely with the technology employed for each activity^16^.

Additionally, AHF can be spatially estimated based on the concept of energy budget closure^17,18^. This method employs measurements of net radiation along with sensible, latent, and ground heat using remotely sensed meteorological data, which is suitable for local-scale studies^19^. Recently, several studies have combined multiple approaches to estimate AHF for large regions^20,21^. For example, Lee *et al*.^22^ built a regression model that used AHF calculated by an inventory-based approach and pollutant emission data to estimate AHF over the entire US. However, generalizing this method to a global scale has proved difficult, because such simulations of AHF are subject to the availability of data from multiple sources. Given the features and limitations of existing approaches, a top-down approach should currently be the most efficient for estimating the distribution of global AHF.

Therefore, the objective of this study is to develop a new long-term (1970–2050) global gridded AHF dataset with fine resolution using a top-down approach based on population density data. The new dataset, including past and future global gridded AHF (PF-AHF), was compared with previous estimates at multiple scales (from the city to global level) for validation.

## Methods

### Data collection

#### Statistical energy consumption data

Annual primary energy consumption data from 1965 to 2016 were obtained from the British Petroleum (BP) Statistical Review of World Energy (hereinafter, BP Review) (https://www.bp.com/). Primary energy sources comprise coal, hydroelectricity, nuclear energy, natural gas, oil, and modern renewables used to generate electricity. The BP Review classified the entire earth into six sub-regions, which include 65 countries and five other regions (Table 1). Based on the BP energy consumption data in 2015, the total energy consumption of the above-mentioned 65 countries counted about 95.8% of the global amount, indicating that these 65 countries are the most significant places for energy consumption and anthropogenic heat release. The energy statistics reported by BP with relatively high reliability have been used in many previous studies^7,23^. In addition, future energy demands reported by the 2018 Reference Case of McKinsey GEP were used to estimate annual growth rates in future energy demand (https://gep.mckinseyenergyinsights.com/).

**Table 1:** List of the countries and regions in six sub-regions as listed in the British Petroleum Statistical Review of World Energy.

Sub-regions in the world	Countries or regions
North America	United States of America, Canada, Mexico
South and Central America	Argentina, Brazil, Chile, Colombia, Ecuador, Peru, Republic of Trinidad and Tobago, Venezuela, other regions
Europe and Eurasia	Austria, Azerbaijan, Belarus, Belgium, Bulgaria, Czech Republic, Denmark, Finland, France, Germany, Greece, Hungary, Ireland, Italy, Kazakhstan, Lithuania, Netherlands, Norway, Poland, Portugal, Romania, Russian Federation, Slovakia, Spain, Sweden, Switzerland, Turkey, Turkmenistan, Ukraine, United Kingdom, Uzbekistan, other regions
Middle East	Iran, Israel, Kuwait, Qatar, Saudi Arabia, United Arab Emirates, other regions
Africa	Algeria, Egypt, South Africa, other regions
Asia Pacific	Australia, Bangladesh, China, India, Indonesia, Japan, Malaysia, New Zealand, Pakistan, Philippines, Singapore, Republic of Korea, Thailand, Vietnam, other regions

#### Global population data

Global gridded population density data representing conditions in 1970, 1980, and 1990 were obtained from the Global Population Density Grid Time Series Estimates, Version 1, while the data for 2000, 2005, 2010, and 2015 were obtained from the Gridded Population of the World, Version 4, Revision 10 Data Sets^24,25^. The two above-mentioned datasets were downloaded from the Socioeconomic Data and Applications Center in NASA’s Earth Observing System Data and Information System (http://sedac.ciesin.columbia.edu/data/sets/browse). These two datasets have a spatial resolution of 30 arc-seconds (approximately 1 km at the equator) and are used to redistribute the total energy consumption within a country or region. The population size data were obtained from the Wittgenstein Centre Data Explorer Version 1.2 that includes the global population projections from 1970 to 2100 for 195 countries worldwide (http://www.wittgensteincentre.org/dataexplorer). The future population sizes during 2020–2050 used in this study were generated according to the medium level scenario of the Shared Socioeconomic Pathways scenarios. The assumptions about future trends in fertility, mortality, and migration are authoritative and were based on scientific input from more than 500 population experts worldwide who responded to an online questionnaire and assessed the validity of alternative arguments as well as the conclusions of intensive discussions at five meta-expert meetings^26,27^. We re-counted the population sizes for the 65 countries and five other regions in the six sub-regions mentioned above to maintain data consistency (Table 1).

#### Other data used for validation

The DMSP/OLS night-time stable light (NSL) data in 2010 with a spatial resolution of 1 km were collected by the US Air Force Weather Agency and obtained from the NOAA’s National Geophysical Data Center (https://ngdc.noaa.gov/eog/download.html). The values of NSL data ranging from 0–63 indicate the night-time light intensities. In general, developed areas with a high gross domestic product (GDP) consume more energy than do developing areas with low GDP. It has been demonstrated that night-time light intensity is positively correlated with GDP, population size, and energy consumption^16,28^. Therefore, the night-time light data could be used as a proxy of AHF to validate our PF-AHF dataset^29–31^.

Global Man-made Impervious Surface (GMIS) data in 2010 with a spatial resolution of 1 km were used to extract the spatial extent of urban area; GMIS data were obtained from the Socioeconomic Data and Applications Center in NASA’s Earth Observing System Data and Information System (http://sedac.ciesin.columbia.edu/data/sets/browse). The values of pixels in the GMIS data ranging from 0 to 100% show the percentage of impervious surface. Examples of impervious surface include roads, parking lots, buildings, driveways, sidewalks, and other manmade surfaces^32^. As an important indicator used to assess the urban environment, impervious surface has been frequently used to reflect the land use/cover change associated with urbanization^33,34^. Based on the study of Voorde *et al*.^34^, we extracted the regions with pixels having a GMIS value larger than 10%, and delineated them as urban areas.

### Methodology

**Fig. 1:**
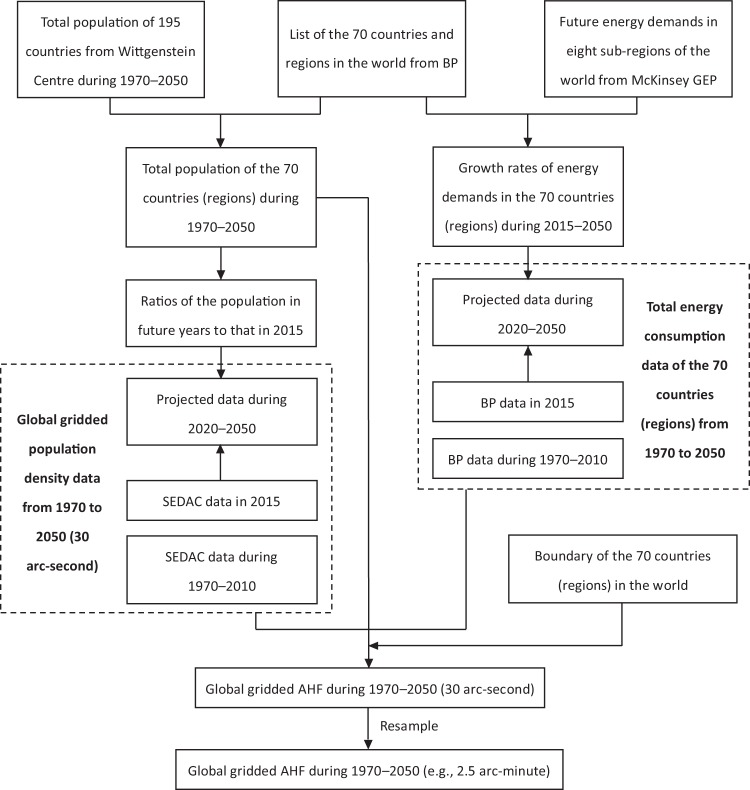
Flow chart of calculations for the past and future global gridded anthropogenic heat flux. AHF, anthropogenic heat flux; BP, British Petroleum; GEP, global energy perspective; SEDAC, Socioeconomic Data and Applications Center.

The flow chart used for developing the PF AHF dataset in this study is presented in Fig. 1. First, we aggregated the total population data of 195 countries from the Wittgenstein Centre to the 70 countries and regions listed in the BP Review. Next, the distribution of future population density (i.e., 2020, 2030, 2040, and 2050) was estimated based on the 2015 population density data from the Socioeconomic Data and Applications Center and ratio of future population size to that in 2015. Second, we calculated the annual growth rates in future energy demand for the eight global sub-regions classified by McKinsey, and then disaggregated them to the above-mentioned 70 countries and regions to estimate future energy consumption for each country and region. Third, based on the global gridded population density data and the population sizes of the 70 countries and regions, the global AHF at 30 arc-seconds was estimated for each target year through redistributing the total energy consumption at the country level. Finally, spatial resolution of the calculated global gridded AHF data was decreased from 30 arc-second to 2.5 arc-minute by resampling in ArcGIS software (ESRI, Redlands, CA, USA).

### Projection of future energy consumption

Based on the future energy demands reported by the GEP, the annual growth rates of the energy demand during 2015–2030 and 2030–2050 were estimated for different regions of the world (Table 2). According to the level of energy consumption for 2015 reported by BP and the growth rates of energy demand mentioned above, we further estimated the energy consumption of 65 countries and five other regions in 2020, 2030, 2040, and 2050. The GEP represents McKinsey’s latest consensus on how the energy transition will unfold, which takes into account both macro- and micro-economic developments (e.g., shifts in electricity demand curves, population, and GDP). It is based on a bottom-up energy demand model, showing energy demand projections for 145 countries, 28 sectors, and 55 fuel types. For each sector and country, a detailed methodology defines how fundamental drivers such as economic growth and population expansion, and key developments such as efficiency improvements and fuel shifts drive the evolution of energy demand. For example, McKinsey adopted a road transport model to calculate fuel demand for vehicles in each country, which covers five vehicle segments (cars, vans and pickups, trucks, buses, and 2- and 3-wheelers) and four distinct elements (vehicle sales, type of powertrain, fuel consumption, and expected annual distance travelled). More details about the GEP model can be accessible online (https://gep.mckinseyenergyinsights.com/the-model/).

**Table 2:** Growth rates of future energy demands in eight sub-regions of the world.

Regions	Primary energy demand (million terajoules)			Growth rate per annum (%)	
	2015	2030	2050	2015−2030	2030−2050
OECD Americas	115	114	106	−0.1	−0.4
OECD Europe	75	71	63	−0.4	−0.6
OECD Asia Pacific	37	37	34	0.0	−0.4
China	124	144	138	1.0	−0.2
India	35	57	89	3.3	2.3
Other Asia countries	41	55	77	2.0	1.7
Africa	33	45	70	2.1	2.2
Rest of World	106	123	142	1.0	0.7

Growth rates of energy demands were calculated based on data reported by the 2018 Reference Case of the McKinsey Global Energy Perspective. Note: OECD, Organization for Economic Co-operation and Development.

### Projection of future population density

In the present study, global population density data in the targeted future years (i.e., 2020, 2030, 2040, and 2050) were estimated based on the two following steps. First, the ratios of the population size in a target year to that in 2015 were calculated for the 70 countries and regions listed in Table 1. Second, global population density in the target year was estimated by multiplying the population density of a given country or region in 2015 by the corresponding population growth ratio. The global population projections from the Wittgenstein Centre Data Explorer Version 1.2 considered the fertility, mortality, and migration of population for each country. In the present study, therefore, the estimated data on future population density also contain information related to country-level population movement. These calculations were conducted in ArcGIS software using Eq. (1):

1 $${\rho }_{grid,i,j}={\rho }_{grid,2015,j}\times \frac{{P}_{total,i,j}}{{P}_{total,2015,j}},$$

where *ρ*_*grid*,*i*,*j*_ indicates the population density of each grid in the country (region) *j* for the target year *i* in person/m^2^, *i* indicates 2020, 2030, 2040, or 2050, *j* indicates one of the 70 countries and regions listed in Table 1, *ρ*_*grid*,*2015*,*j*_ indicates the population density of each grid in 2015 in the country (region) *j* in person/m^2^, *P*_*total*,*i*,*j*_ indicates total population of the country (region) *j* in the target year *i* in number of people, *P*_*total*,*2015*,*j*_ indicates total population of the country (region) *j* in 2015 in number of people. *P*_*total*,*i*,*j*_ was obtained from the Wittgenstein Centre Data Explorer Version 1.2 according to the medium Shared Socioeconomic Pathways scenarios (http://www.wittgensteincentre.org/dataexplorer).

### Estimation of global gridded AHF

This study adopted the top-down inventory-based approach and current national boundaries to redistribute the total energy consumption within each country or region. Based on previous studies^3,7^, we assumed that all consumed energy was eventually converted into heat. Moreover, the delay between energy use and its conversion to heat was ignored^35^. Thus, the gridded AHF of the world could be calculated in ArcGIS software using Eq. (2):

2 $$AH{F}_{i,j}=\frac{{E}_{total,i,j}\times \frac{{P}_{grid,i,j}}{{P}_{total,i,j}}\times C}{{S}_{grid,j}\times {t}_{i}},$$

where AHF_*i*,*j*_ means gridded anthropogenic heat flux induced by energy consumption in the country (region) *j* for the target year *i* in W/m^2^, *i* indicates 1970, 1980, 1990, 2000, 2005, 2010, 2015, 2020, 2030, 2040, or 2050, *j* indicates one of the 70 countries and regions listed in Table 1, *E*_*total*,*i*,*j*_ indicates total energy consumption of the country (region) *j* in the target year *i* in tonne oil equivalent (toe), *P*_*grid*,*i*,*j*_ means the total population of each grid in the country (region) *j* for the target year *i* in number of people, *P*_*total*,*i*,*j*_ indicates total population of the country (region) *j* in the target year *i* in number of people, *S*_*grid*,*j*_ represents the area of each grid in the country (region) *j* in m^2^, *t*_*i*_ represents the total time of the target year *i* in s, and *C* represents the energy conversion coefficient, 1 toe = 42 × 10^9^ J.

Because the ratio of *P*_*grid*,*i*,*j*_ to *S*_*grid*,*j*_ equals to the population density of a grid in region *j* for the target year *i*, Eq. (2) can be represented by Eqs (3), (4), and (5):

3 $$AHF={M}_{i,j}\times {\rho }_{grid,i,j},$$

4 $${M}_{i,j}=\frac{{E}_{total,i,j}\times C}{{P}_{total,i,j}\times t},$$

and

5 $${\rho }_{grid,i,j}=\frac{{P}_{grid,i,j}}{{S}_{grid,j}},$$

where *ρ*_*grid*,*i*,*j*_ indicates the population density of each grid in region *j* for the target year *i*, in person/m^2^.

After data pre-processing, two main steps were conducted to produce the global gridded AHF data. First, based on the Eq. 3, Micro Excel 2010 software was used to calculate the parameter *M*_*i*,*j*_ for each country or region. Second, the Raster Calculator in ArcGIS 10.2 software was used on the gridded AHFs in a given country or region to calculate the gridded population density data multiplied by the corresponding *M*_*i*,*j*_.

**Fig. 2:**
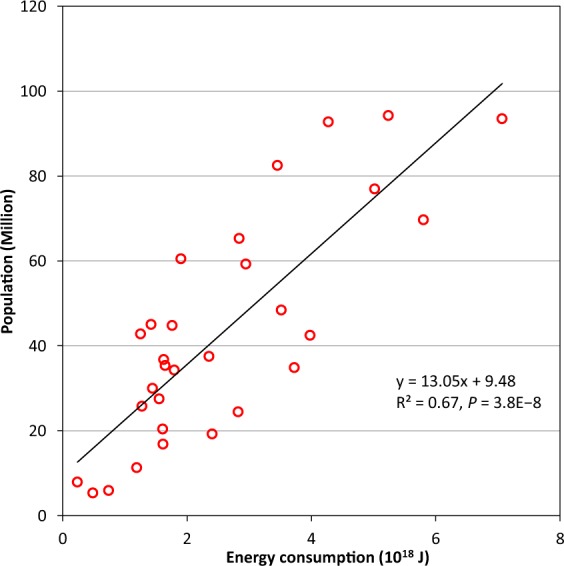
Relationship between energy consumption and population in 2005 for the 30 administrative regions of China. Significance was determined using a two-tailed confidence t-test in SPSS 19.0 software (P < 0.05).

In this process, we adopted population data to redistribute the total energy consumption into each grid in a country or region. Because of the lack of detailed information related to energy consumption (e.g., industrial distribution, energy-use type, and per-capita energy consumption in different areas and periods), we had to assume that each person consumes the same amount of energy in a given country. This assumption may lead to some uncertainties at a local scale because it fails to consider specific socio-economic factors. In this study, we analysed the relationship between energy consumption and population size based on the statistics from 30 provinces and autonomous regions/cities (hereinafter, administrative regions) of China (http://www.stats.gov.cn). The Macao Special Administrative Region, Hong Kong Special Administrative Region, Tibet and Taiwan were not considered because of the limitation of statistical data. Figure 2 shows that a significantly positive correlation exists between energy consumption and population size (*R*^2^ = 0.67, *P* < 0.001). The result indicates that our method and the assumptions used for redistributing total energy consumption at the country level are somewhat reasonable, and can reflect the variations in energy consumption at least at the provincial scale.

### Validation of PF-AHF data

Evaluating AHF data with real measurements spatially has proved difficult because of the difficulties involved in measuring this type of heat flux^9,36^. Therefore, the PF-AHF data were verified by correlation and comparison analyses with the help of other datasets. First, we analysed the spatial distribution of AHF based on the new gridded database, and compared it with the DMSP/OLS night-time light image data at a global scale. Correlation analysis between averaged AHFs and averaged night-time light intensities was conducted for the 32 administrative regions of China by SPSS 19.0 software (the Macao and Hong Kong Special Administrative Regions were excluded because of the small area they include). Night-time light intensity has been frequently used as a proxy for anthropogenic heat release^28–30^, indicating that the correlation between AHFs and night-time light intensities can be indirectly used to evaluate the accuracy of PF-AHF data. Second, using a same spatial resolution (2.5 × 2.5 arc-minute), we analysed the difference in annual mean AHF over the entire planet between the PF-AHF data and a previous AHF dataset produced by Flanner^2^. The averaged AHFs of the 100 largest cities in the world ranked by population size in 2010 were estimated based on both the PF-AHF and Flanner’s^2^ datasets, independently. Then, correlation analysis between the PF-AHF and Flanner’s dataset was conducted for these 100 largest cities. Third, we compared the averaged AHF of some cities of the world reported in previous studies with the AHF calculated using the PF-AHF data. Urban areas of a given city were determined based on the impervious surfaces extracted from GMIS data.

Moreover, a cross-validation method was used to evaluate uncertainties of our approaches for calculating future AHF data. First, we assumed this year is year 2000. Next, based on the population density data in 2000 and the population size projections in 2000, 2005, 2010, and 2015, we recalculated the distribution of ‘future’ population density (i.e., 2005, 2010, and 2015) using Eq. (1). Second, based on the BP energy consumption data in 2000 and the GEP energy demands in 2030, we recalculated the annual growth rate of the energy demand during 2000–2030 and estimated the ‘future’ energy consumption data (i.e., 2005, 2010, and 2015). Third, based on these recalculated ‘future’ data and the Eqs (3) and (4), global gridded AHF data in 2005, 2010, and 2015 were projected for test (i.e., test data). Finally, based on the root mean squared error (RMSE) and coefficient of determination (R^2^)^37^, the relationship between the measured data (i.e., PF-AHF data) and the test data was analysed for the selected 100 largest cities in the world. Additionally, we resampled the test data in 2010 to varied spatial resolutions (i.e., 30 arc-second, 1 arc-minute, 2 arc-minute, 3 arc-minute, …, 30 arc-minute). Then, the mean AHFs in 2010 for the selected 32 administrative regions of China were estimated based on both the measured data with a spatial resolution of 30 arc-second and the test data with different spatial resolutions, independently. Based on the RMSE and R^2^ between the two kinds of estimations of the 32 administrative regions in 2010, we assessed the uncertainties caused by different spatial resolutions for our PF-AHF data in future years.

## Data Records

The PF-AHF dataset included estimated or predicted annual mean AHF data for 1970, 1980, 1990, 2000, 2005, 2010, 2015, 2020, 2030, 2040, and 2050. The PF-AHF dataset had two levels of spatial resolution (30 arc-second and 2.5 arc-minute). The uploaded data at the figshare repository were tagged in image file format and included global gridded AHF imagery for 1970, 2015, and 2050^38^. These data can be processed by ArcGIS software, etc. The values of grids represented the annual mean heat flux induced by energy consumption, in W/m^2^.

## Technical Validation

### Spatiotemporal patterns of AHF and validation using night-time light data

**Fig. 3:**
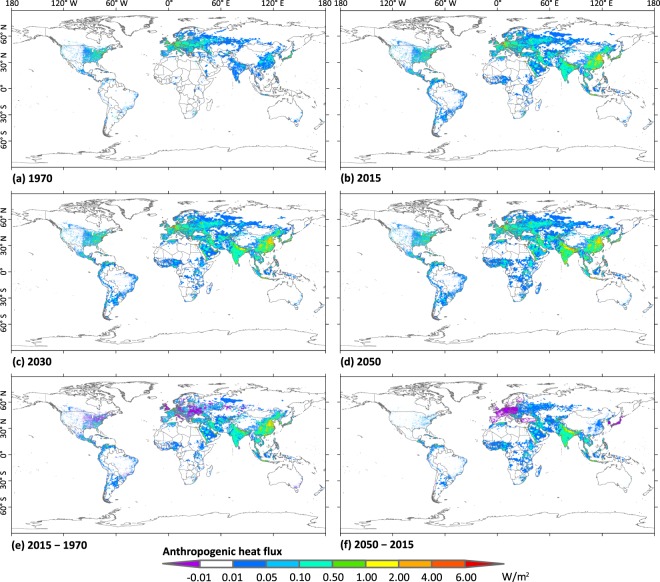
Spatiotemporal changes in the annual mean anthropogenic heat flux (AHF) based on the past and future global gridded AHF dataset. (**a**) shows spatial distribution of AHF in 1970. (**b**–**d**) are same as (**a**) but for 2015, 2030, and 2050, respectively. (**e**) shows the difference in AHF between 1970 and 2015 and (**f**) shows the difference in AHF between 2015 and 2050.

Based on energy consumption and global land area statistical data, the globally averaged terrestrial AHFs were estimated to be 0.05, 0.13, 0.15, and 0.16 W/m^2^ in 1970, 2015, 2030, and 2050, respectively, although these data varied among different continents (Fig. 3). While Asia experienced the greatest increase of AHF in the period of 1970–2015, some regions of Europe and US showed a decrease in AHF (Fig. 3e). In the future, grids with a substantial change in AHF are predicted to mostly be located in the developed and developing regions of the planet (Fig. 3f).

**Fig. 4:**
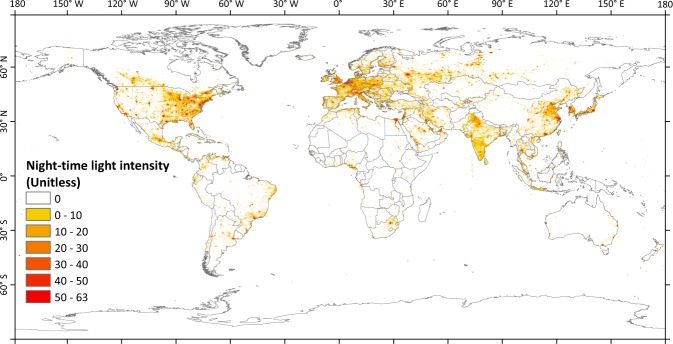
Spatial distribution of night-time light intensity in 2013.

**Fig. 5:**
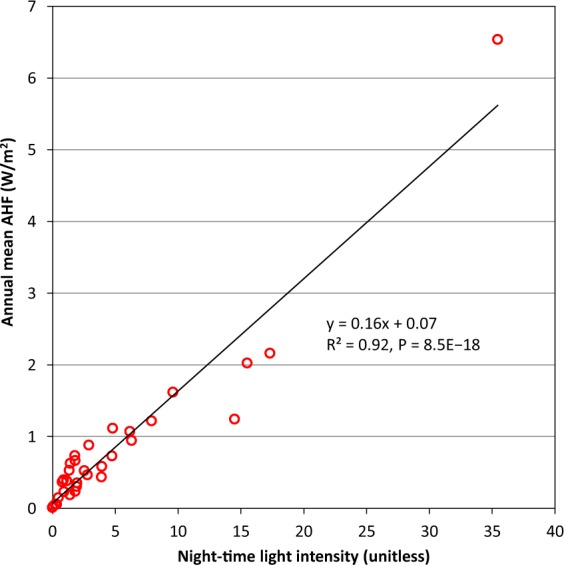
Relationship between averaged anthropogenic heat flux (AHF) and averaged night-time light intensity in 2005 for the 32 administrative regions of China. The significance was determined using a two-tailed confidence t-test (P < 0.05).

Overall, the AHFs induced by energy consumption were larger in the Northern Hemisphere (especially in 20–60°N) than the Southern Hemisphere in the past few decades. This could be one of the reasons for the warming occurring over mid- and high latitudes in Northern Hemisphere^5^. Additionally, the spatial distribution of AHF was consistent with the night-time light intensity at a global scale (Figs 3 and 4). At a provincial scale, the correlation between averaged AHF and night-time light intensity was significant (*P* < 0.001; Fig. 5), which is consistent with the report of Chen *et al*.^28^. All of these results imply that the annual mean AHF data are consistent with the distribution, intensity, and magnitude of human activities; that is, a large number of cities located in the middle latitudes of the Northern Hemisphere consume a great deal of energy^5,9,10^.

### Comparison of the differences between PF-AHF data and Flanner’s^2^ AHF data

In the present study, the global AHF dataset calculated by Flanner^2^ with a resolution of 2.5 × 2.5 arc-minutes that was based on population density data was compared with our PF-AHF data^2^. Flanner’s data had been developed based on historical energy consumption records for 2005, the EIA energy-use projections during 2006–2040 (high economic growth case), and the assumed growth rate of energy-use during 2040–2100 (i.e., 2%). First, we analysed the difference in annual mean AHF between the PF-AHF data and Flanner’s data in 2005 to validate the accuracy of the PF-AHF data. Second, we compared the PF-AHF data with Flanner’s data in 2015 and 2030 to evaluate the uncertainties resulted from the use of different projections of energy consumption.

**Fig. 6:**
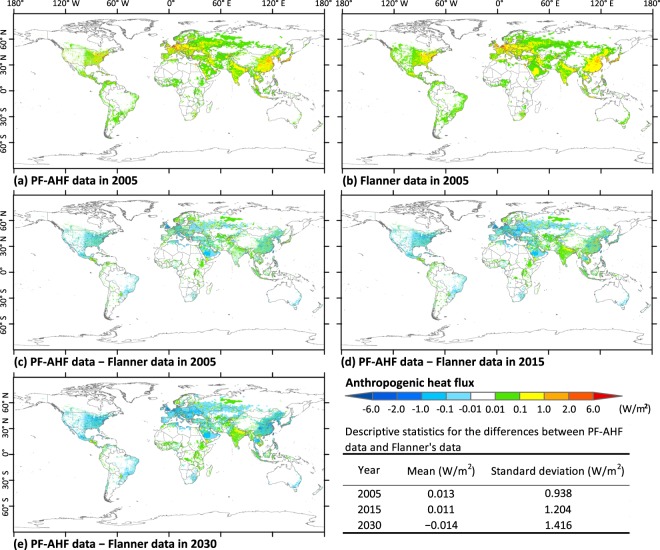
Comparison of the past and future global gridded anthropogenic heat flux (PF-AHF) data with Flanner’s data (2.5 × 2.5 arc-minute). Top row represents spatial distribution of the annual mean anthropogenic heat flux (AHF) for 2005 based on (**a**) the PF-AHF data and (**b**) Flanner’s^2^ data. (**c**) shows the spatial difference in annual mean AHF between the PF-AHF data and Flanner’s^2^ data in 2005. (**d**,**e**) are same as (**c**) but for 2015 and 2030, respectively.

The distribution of mean annual AHF from the PF-AHF data for 2005 agreed well with Flanner’s data (Fig. 6a,b). Differences in measured AHF between the two datasets were mostly between −0.1 and 0.1 W/m^2^, which is very small at a global scale (Fig. 6c). Pixels with an extremely slight difference between the two datasets (−0.01 to 0.01 W/m^2^) were mainly distributed in regions known to consume little energy. In contrast, pixels with relatively large differences (>2 W/m^2^ or < −2 W/m^2^) were mainly distributed in urban areas known to consume a great deal of energy. The spatial patterns of the differences in AHF between the PF-AHF data and Flanner’s data in 2015 and 2030 were consistent with that in 2005 (Fig. 6c–e). However, the regional differences in AHF between the two datasets in 2015 and 2030 were larger than those in 2005. For example, in India, more pixels with a difference ranging from 0.01 to 0.1 W/m^2^ were observed in 2015 than those observed in 2005; in China, more pixels with a difference ranging from −1.0 to −0.1 W/m^2^ were observed in 2030 than those observed in 2005. Descriptive statistic shows that means of the difference in pixel value between the two datasets were 0.013, 0.011, and −0.014 W/m^2^ in 2005, 2015, and 2030, respectively (Fig. 6). This indicates that the historic AHF data may have been underestimated by Flanner^2^ relative to this study, while he may have overestimated the AHF data for future period. Moreover, standard deviations of the differences in pixel values between the two datasets indicate that the difference between the two datasets in 2030 has larger spatial variability than that in 2005 and 2015.

**Fig. 7:**
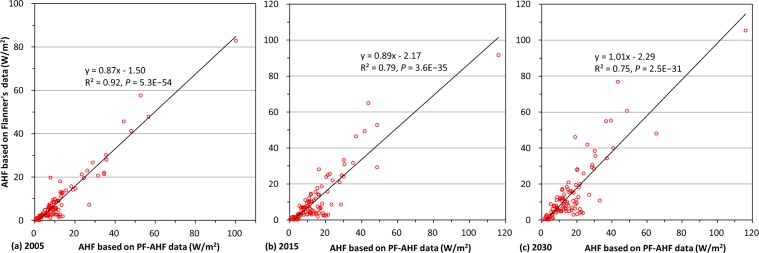
Relationship between the past and future global gridded anthropogenic heat flux (PF-AHF) data and Flanner’s data based on the annual mean anthropogenic heat flux (AHF) of the 100 largest cities in the world. (**a**) shows the relationship between PF-AHF data and Flanner’s^2^ data in 2005. (**b**,**c**) are same as (**a**) but for 2015 and 2030, respectively. The significance was determined using a two-tailed confidence t-test (P < 0.05).

Figure 7 shows that a significantly positive correlation exists between the PF-AHF and Flanner’s datasets based on the annual mean AHF of the 100 largest cities in the world (*P* < 0.001). Moreover, based on the annual mean AHF of the 100 cities, correlation coefficients between the PF-AHF data and Flanner’s data were 0.96, 0.89, and 0.87 in 2005, 2015, and 2030, respectively (Fig. 7). This indicates that the agreement between the PF-AHF and Flanner’s datasets in 2005 was closer than that in 2015 and 2030. The correlation between the PF-AHF and Flanner’s datasets in 2030 was the lowest, which is consistent with the result based on Fig. 6.

Differences between the two datasets were largely attributed to differences in energy consumption data and variations in the population density data used in this study and in Flanner^2^. For example, energy data used in Flanner’s study did not include renewable energy, which features an increasing percentage over time in recent years^2^. This may be one reason why the mean of the PF-AHF data was larger than the mean of Flanner’s data in 2005 and 2015. Moreover, the energy-use projections of EIA used in Flanner’s study were based on a high economic growth case^2^, which differs from the energy demands of GEP used in the present study. This may be the main reason why the mean of the PF-AHF data was smaller than the mean of Flanner’s data in 2030. Decreasing the pixel resolution of our data for comparison may also lead to a certain amount of bias in those areas that lie close to large water bodies^9^.

### Validation of PF-AHF data using the results reported by previous studies

For comparison, we selected some cities where other researchers have analysed the anthropogenic heat release in recent years. Table 3 shows that AHFs have been estimated at 10–90 W/m^2^ for most cities based on the PF-AHF data, findings that are comparable with previous findings. However, some differences in the estimates between the present and previous studies exist. With three specific cities located in Eastern China for example, the AHFs of the cities of Beijing, Shanghai, and Taiyuan were estimated to be 8.4, 15.5, and 13.5 W/m^2^, respectively, based on the PF-AHF database. The above three estimates differ from the corresponding previous results listed in Table 3 to some extent. Lu *et al*.^39^ reported that the AHFs in Eastern China were mostly between 0.5 and 20 W/m^2^, indicating that the estimates based on the PF-AHF database are in a reasonable range. The difference between the current estimates based on PF-AHF database and previous results may be related to the differences in the spatial ranges used to delineate the cities as well as differences in data sources and methodologies.

**Table 3:** Some examples of anthropogenic heat flux (AHFs) in urban areas of selected cities estimated by the present and previous studies.

Cities	AHF in this study (W/m^2^)	The results in previous studies		
		AHF (W/m^2^)	Method	Reference
Beijing, China	8.4 in 2010	14.55 in 2011	Bottom-up	^40^
Shanghai, China	15.5 in 2010	19 in 2010	Top-down	^16^
Taiyuan, Shanxi, China	13.5 in 2010	7.8 in 2010		
Incheon, Republic of Korea	52.2 in 2000	53 in 2002	Top-down and Bottom-up	^47^
Seoul, Republic of Korea	87.5 in 2000	55 in 2002		
Tokyo, Japan	52.8 in 2015	41.4 in 2013	Top-down	^8^
Houston, TX, US	12.2 in 2005	14.6 in summer, 2005	Statistical regression	^22^
New York, NY, US	54.7 in 2005	48 in 2005	Top-down	^2^
Phoenix, AZ, US	11.2 in 2010	13 in summer, 2012	Bottom-up	^16^
Montreal, QC, Canada	34.8 in 2010	35 in winter, 2007–2009	Energy budget closure	^19^
Sao Paulo, Brazil	13.6 in 2005	20 in 2004–2007	Bottom-up	^48^
Sydney, Australia	19.9 in 2010	13–59.3 in 2007–2009	Bottom-up	^4^
Johannesburg, South Africa	11.8 in 2010	4.52 ± 7.87 in 2011	Top-down	^49^
Basel, Switzerland	33.5 in 2000	20 in 2001–2002	Energy budget closure	^18^
Helsinki, Finland	10.7 in 2010	13 in 2007–2010	Energy budget closure	^17^
London, UK	26.2 in 2015	16–24 in 2015	Top-down	^9^
Toulouse, France	7.9 in 2000	7.2 in 2000	Bottom-up	^7^

In addition, we found that our estimates for the cities of Houston, TX, USA, Phoenix, AZ, USA, and Montreal, QC, Canada were smaller than corresponding results reported in previous studies. This occurred because the estimated AHFs of Houston, Phoenix, and Montreal in previous studies focused on summer and winter, which are generally larger than in spring and autumn^9,40^. Allen *et al*.^9^ demonstrated that the AHF peaked from December to February in the Northern Hemisphere, while the AHFs in July and August were also high. Regional climates greatly affect the intensity of heat releases^41^. During winter in the Northern Hemisphere, apart from the energy consumption for normal production, the cold climate causes a very large increase in energy consumed for residential heating^42^. The relatively high AHF during summer in the Northern Hemisphere is mainly caused by the use of cooling systems^42^. For this reason, most previous studies of anthropogenic heat release have focused on summer and winter. However, monthly energy consumption data are difficult to obtain covering large-scale regions. Flanner^2^ adopted several latitudinally dependent equations to explore seasonal AHF cycles, and provided a selection for research results on projected future intra-annual variations of global AHF.

### Validation of PF-AHF data using a cross-validation method

Using the data before 2000 and the approaches for calculating future AHF data in this study, we projected the AHF data in 2005, 2010, and 2015 for test. Based on these test data, the average AHF of the 100 largest cities in the world in 2005 was calculated to be about 11.44 W/m^2^, which is slightly larger than the corresponding measurement based on the PF-AHF data (Table 4). However, the test data in 2010 and 2015 were smaller than the corresponding measurements overall. These differences between the two abovementioned data were mainly caused by the use of the different energy consumption data. While the measured data were calculated using the energy consumption records, the test data were calculated using the energy consumption projections based on the average annual growth rate of the energy demand during 2000–2030. This growth rate was higher than the reality in the early period but lower than the reality in the late period. Moreover, while RMSE between the test data and the measured data increased from 2.80 W/m^2^ in 2005 to 5.12 W/m^2^ in 2015, their R^2^ decreased from 0.97 in 2005 to 0.88 in 2015. These imply that the differences of AHF between the projections and the actual situation would increase with time. However, there were significant correlations between the test data and the measured data in 2005, 2010, and 2015, implying certain reliability of the projected AHF data based on the approaches used in this study.

**Table 4:** Relationship between the test data and the measured data based on the annual mean anthropogenic heat flux (AHF) of the 100 largest cities in the world.

Year	Averaged AHF (W/m^2^)		RMSE (W/m^2^)	R^2^	P-value
	Test data	Measured data			
2005	11.44	11.07	2.80	0.97	4.4E−76
2010	12.23	14.12	3.83	0.95	7.1E−66
2015	14.35	14.96	5.12	0.88	2.9E−46

The test data in 2005, 2010, and 2015 were from the projected AHF data based on the data before 2000. The measured data were from the past and future global gridded AHF (PF-AHF) data. Note: RMSE, root mean squared error; R^2^, coefficient of determination.

**Fig. 8:**
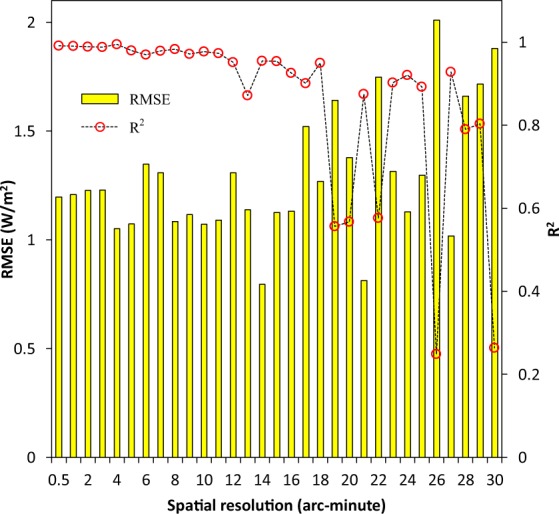
Relationship between the measured data in 30 arc-second and the test data in varied spatial resolutions based on the annual mean anthropogenic heat flux (AHF) of the 32 administrative regions of China in 2010. The measured data were from the past and future global gridded AHF (PF-AHF) dataset. The test data were from the projected AHF data based on the data before 2000. RMSE, root mean squared error; R^2^, coefficient of determination.

Fig. 8 shows that RMSE between the measured data and the test data gradually increased with the decrease in spatial resolution of the test data. Contrarily, R^2^ between the two abovementioned data gradually decreased with the decrease in spatial resolution of the test data. Moreover, the least RMSE between the measured data and the test data was found when the spatial resolution of the test data was 14 arc-minute. The largest R^2^ between the two data was found when the spatial resolution of the test data was 4 arc-minute. Overall, the biases between the measured data and the test data were relatively small and their correlations were relatively strong when the spatial resolution of the test data was in the ranges of 4~5 arc-minute and 8~11 arc-minute. These implies that the spatial resolutions of 4~5 arc-minute and 8~11 arc-minute may be more appropriate for the future time series of the PF-AHF dataset.

## Usage Notes

The Fifth Assessment Report of the Intergovernmental Panel on Climate Change showed that the radiative forcing of CO_2_ has increased by 0.27 (0.24–0.30) W/m^2^ in the past decade^43^. This implies that the average radiative forcing of annual released CO_2_ was approximately 0.027 W/m^2^ during this period, which is comparable to the current global annual mean AHF (i.e., 0.03 W/m^2^)^7^. Anthropogenic heat release has influenced changes in climates globally, especially for the Northern Hemisphere^5,44^. In densely populated areas, AHFs have been much larger than the global average level, and this forcing is expected to continue to grow in developing regions^5,45^. Thus, incorporating anthropogenic heat into global climate models could help us to improve the performance of simulations of surface climate warming^11^.

The PF-AHF data predict a weakening of AHF at some regions in the coming decades that is closely related to the projected consumption of energy reported by McKinsey in the present study. The changes in the quantity of energy consumed can be induced by economic development, shifting economic structure, changes in energy consumption type, and improved energy use efficiency^46^, which have been considered by McKinsey (https://gep.mckinseyenergyinsights.com/). However, the single existing global gridded dataset associated with future AHF, Flanner’s data, was built on an early energy-use projection of EIA during 2006–2040 (high economic growth case) and a simple assumption that the growth rate of energy consumption after 2040 will be 2% per year for all countries^2^. Evidently, Flanner’s data from 2006 to 2015 have been out of date, and his data after 2015 may contain large uncertainties^2^. Correlation analysis showed that the correlation between the two datasets in 2015 and 2030 was weaker than that in 2005, implying the necessity of updating global AHF data based on new statistics and predictions of energy consumption. When compared with Flanner’s data^2^, our data are more objective in describing the global distribution of future AHFs. The accuracy of our PF-AHF data in future period was also confirmed by the cross-validation method. Additionally, the history time series of the PF-AHF database is longer than that of all previous datasets^2,7^, providing an advantage in simulating long-term climatic effects using the dataset developed in this study.

However, the PF-AHF database may still include some uncertainties. For example, we assumed that all consumed energy was eventually converted into heat, and the delay between energy use and its conversion to heat was ignored. Since some energy that is consumed will be stored by buildings and converted to other forms of energy^35^, this assumption is somewhat unreasonable. As a result, the present study may overestimate the amount of anthropogenic heat produced. This uncertainty also existed in previous studies because of the difficulties in observing and estimating the conversion efficiency from fuel to heat^11^. Another uncertainty is induced by the assumption that each person consumes the same amount of energy in a given country when redistributing total energy consumption in each country. As we know, energy consumption rates may be different between different regions in a large country because of unbalanced regional economic development (e.g., China). However, specific information about energy consumption such as industrial distribution, energy-use type, and per-capita energy consumption in different areas and periods is difficult to obtain for each country of the world. In order to produce a global gridded AHF dataset with long-term time series, we have to use the country-level statistics to conduct this work. Figure 2 indicates that our method and assumption for redistributing the total energy consumption at a country level are somewhat reasonable. Validation results imply that the PF-AHF data can well reflect the spatiotemporal variation of AHF globally. However, it still should be noted that the above-mentioned uncertainties may be prominent in describing local distribution of AHF, which may lead to a certain biases in simulated results based on climate models, and need to be improved in the future. Thus, selecting appropriate spatial resolution of the AHF datasets for researches at different scales is necessary. This study found that the spatial resolution at the ranges of 4~5 arc-minute and 8~11 arc-minute may be better for the provincial-scale researches using the future time series of the PF-AHF dataset.

Moreover, the use of different energy consumption datasets may lead to some divergence in the estimation of AHF. For instance, we noted that total energy consumption in Singapore was reported at about 81.0 million toe in 2015 in the BP Review (https://www.bp.com/), while it was 17.1 million toe in the 2017 US Energy Information Administration Global Energy Dataset (https://www.eia.gov/). Although these two datasets are frequently used in studies associated with energy consumption and show a high level of correlation, the differences in small areas may lead to strikingly different AHFs in the same grid. These differences in AHF may further lead to different results when simulating the climatic effects of those AHFs^1,4,45^.

Overall, multiple validations revealed that the PF-AHF data were consistent with the night-time light data and the levels of AHF reported in previous studies. The relatively long time series and fine spatial resolution of this new global gridded AHF dataset could provide needed support for the simulation of climate change induced by anthropogenic heat release.

## ISA-Tab metadata file

Download metadata file

## Data Availability

We did not use any custom coding in the process of producing the PF-AHF dataset. In this study, Microsoft Excel and ArcGIS software were employed to process all the data such as the future population density data and the gridded AHF intensity. ArcGIS software was also used to resample the PF-AHF data and draw the figures.
